# Journal Impact Factor Shapes Scientists’ Reward Signal in the Prospect of Publication

**DOI:** 10.1371/journal.pone.0142537

**Published:** 2015-11-10

**Authors:** Frieder Michel Paulus, Lena Rademacher, Theo Alexander Jose Schäfer, Laura Müller-Pinzler, Sören Krach

**Affiliations:** Social Neuroscience Lab, Department of Psychiatry and Psychotherapy, University of Lübeck, Lübeck, Germany; Duke University Medical Center, UNITED STATES

## Abstract

The incentive structure of a scientist’s life is increasingly mimicking economic principles. While intensely criticized, the journal impact factor (JIF) has taken a role as the new currency for scientists. Successful goal-directed behavior in academia thus requires knowledge about the JIF. Using functional neuroimaging we examined how the JIF, as a powerful incentive in academia, has shaped the behavior of scientists and the reward signal in the striatum. We demonstrate that the reward signal in the nucleus accumbens increases with higher JIF during the anticipation of a publication and found a positive correlation with the personal publication record (pJIF) supporting the notion that scientists have incorporated the predominant reward principle of the scientific community in their reward system. The implications of this behavioral adaptation within the ecological niche of the scientist’s habitat remain unknown, but may also have effects which were not intended by the community.

## Introduction

In a time in which money is the dominating remunerative incentive for guiding behavior on the labor market, journal publications have assumed the role of the new “currency” for scientists. Decisions about faculty positions, contracts, salaries or grant applications depend on a scientist’s publication record [1]. Besides the total number of publications, their quality is a decisive prerequisite for academic success [2]. Evaluating the quality of a manuscript is a complex undertaking, but in practice has been increasingly linked to the journal impact factor (JIF, see Nature Editorial on “Not-so- deep impact”) [2,3]. Basically, the JIF is defined as the year’s average number of citations per paper published in a specific journal during the preceding 2 years [4]. Taking the JIF as *the* measure of scientific quality has been intensely criticized, and there is a lively and ongoing debate about how the JIF compromises academia in general [5–9]. Despite the debate on the validity of the JIF as the main criterion for scientific quality and its impact on science (see S1 Note; Criteria of “scientific excellence”), its use is growing, and there is empirical evidence that it influences decision-making in academia [2]. Indeed, this relatively new measure has become deeply entrenched in science and in the evaluation of academic success, be it on a personal, institutional, or national level (*Nature* Editorial 2005). Thus, it seems a logical adaptation of the prevailing principle in science to treat the JIF as a value of a publication just as numbers represent the value of a bill.

The JIF has the potential to deeply change the motivational forces of scientists. In a world where the JIF determines the value of a publication successful goal-directed behavior requires knowledge about the JIF and the ability to make use of this knowledge when potential publications are within reach. However, it remains unknown how scientists have incorporated the JIF into their reward circuitry and adapted their behavior in order to match the affordances for “survival” in academia.

The process of how reward opportunities in the environment shape our behavior is conceptually divided into two distinct phases: an appetitive phase, in which behavior is adjusted to achieve the expected reward, and a consummatory phase, in which the reward is consumed and stimulus-reward contingencies develop [10]. The nucleus accumbens (NAcc), part of the ventral striatum, is known to influence our behavior. Animal studies have demonstrated that dopaminergic nerve cells that project from the midbrain into the NAcc encode reward prediction error signals. That is, they show activations in response to unpredicted and novel rewards. However, if the reward is preceded by a predictive cue, the firing responses shift towards the predictive cue, reflecting reward anticipation [11]. Thus, based on learned associations, dopamine release into the NAcc alerts the organism that a motivationally relevant event is within reach. Further research has shown that the dopamine release signals the value of a potential outcome [12–14]. This is supported by functional neuroimaging studies in humans, which provide evidence that the NAcc response increases with stronger reward value of the anticipated stimulus. NAcc activity has been shown for a variety of anticipated rewards such as money [15,16], pleasant taste [17], verbal praise [15], and appealing faces [18,19], and it has been suggested to represent a ‘common currency’ for the valuation of different reward types [20]. Thus, NAcc activity represents the relative personal value of an anticipated reward, with a key role in addiction and craving behavior [21].

The (publication) life of a scientist maps very well to the reward cycle and we, speaking as scientists, spend the vast majority of time in the appetitive phase. We collect data, apply for grants, and write manuscripts with a significant probability that our research will not bring the desired breakthrough. In this process scientists need to cope with the craving for publication and the urge for the insecure investments to be appreciated accordingly. Here, we present original data showing the contribution of the JIF and the anticipation of a visible publication in a high-impact journal to motivate such behavior. In addition to the JIF as an indicator of the quality of a publication, the authorship position has also been regarded as an important indicator for the evaluation of scientific impact [22] and influences the likelihood of becoming principle investigator [2]. Thus, having a first authorship on a publication is usually associated with greater value, and both dimensions, the JIF and the authorship position, should modulate the scientist’s reward signal and its behavior in the prospect of publication.

Using functional magnetic resonance imaging, we examined the brain activity of *N* = 18 neuroscientists during the anticipation of their own publication. In two consecutive studies, we adapted the classic monetary incentive delay task [16]. Moreover, besides money, we also presented potential journal publications of the participating neuroscientist as rewards for successful behavioral responses. In study one, we manipulated the JIF of the anticipated publication in three steps from low (e.g. NeuroReport, JIF = 1.64), to medium (e.g. NeuroImage, JIF = 6.13), to high (e.g. Nature Neuroscience, JIF = 14.98). In study two, we kept the JIF constant at a high level but varied the authorship position, presenting personalized publications with the participant as either first author or co-author.

## Materials and Methods

### Ethics Statement

We confirm that the research has been conducted in compliance with the ethical guidelines of the American Psychological Association (APA). All participants gave their written informed consent in accordance with guidelines approved by the Ethics Committee of the Philipps-University Marburg, Germany, at the Local Faculty of Medicine (protocol number: 48/14). The Ethics Committee of the Philipps-University Marburg has specifically approved this study.

### Participants

From the initially contacted *N* = 35 eligible neuroscientists at the Department of Psychiatry, Department of Psychology, or Department of Neurology at Marburg University nineteen neuroscientists agreed to participate in this study. One participant was excluded from further analyses due to excessive sleepiness during the whole task. Notably, this was the only neuroscientist with a permanent position. The remaining 18 participants (9 female, aged 25–42 years, *M* = 30.67, *SD* = 4.64) had spent *M* = 5.19 years in science (range 0.5–12 years, *SD* = 2.92) and had an average personal journal impact factor (pJIF) of *M* = 4.08 (range 2.00–5.80 pJIF, *SD* = 1.11). Each individual’s pJIF was extracted based on the work of von Dijk et al. (2014) via the website www.pipredictor.com. Because three participants did not publish in a journal with a JIF at the time of the experiment the pJIF was available for 15 participants.

### Stimuli, Experimental Design, and Procedure

All participants who took part in the study completed two experimental paradigms. For both paradigms the classic “monetary incentive delay” (MID task, [16]) was adapted and instead of money, the title pages of potential publications of the participating neuroscientists were used as incentives (in the following termed “publication incentive delay”, PID; e.g. “*Genetic risk status predicts therapy outcome in major depression*”). Similar to using different amounts of money for the MID, PID incentives comprised personalized title pages of potential neuroscience journal publications with different journal impact factors (JIF). These title pages were designed according to the layout and typography of the title pages of actual articles in a specific journal (e.g. “Nature Neuroscience”). To create plausible incentives for the individual participants the titles and co- authors of the manuscripts were replaced to fit the research scope and the co-author portfolio of the participant. In order to so, participants had to send in a list of seven keywords and up to five potential co-authors together with their affiliations several weeks prior to the experiment. Based on this information, plausible manuscript titles (*N* = 100 for each participating neuroscientist) and co-authors were designed and then inserted in the template in the style of the specific journal.

**Fig 1:**
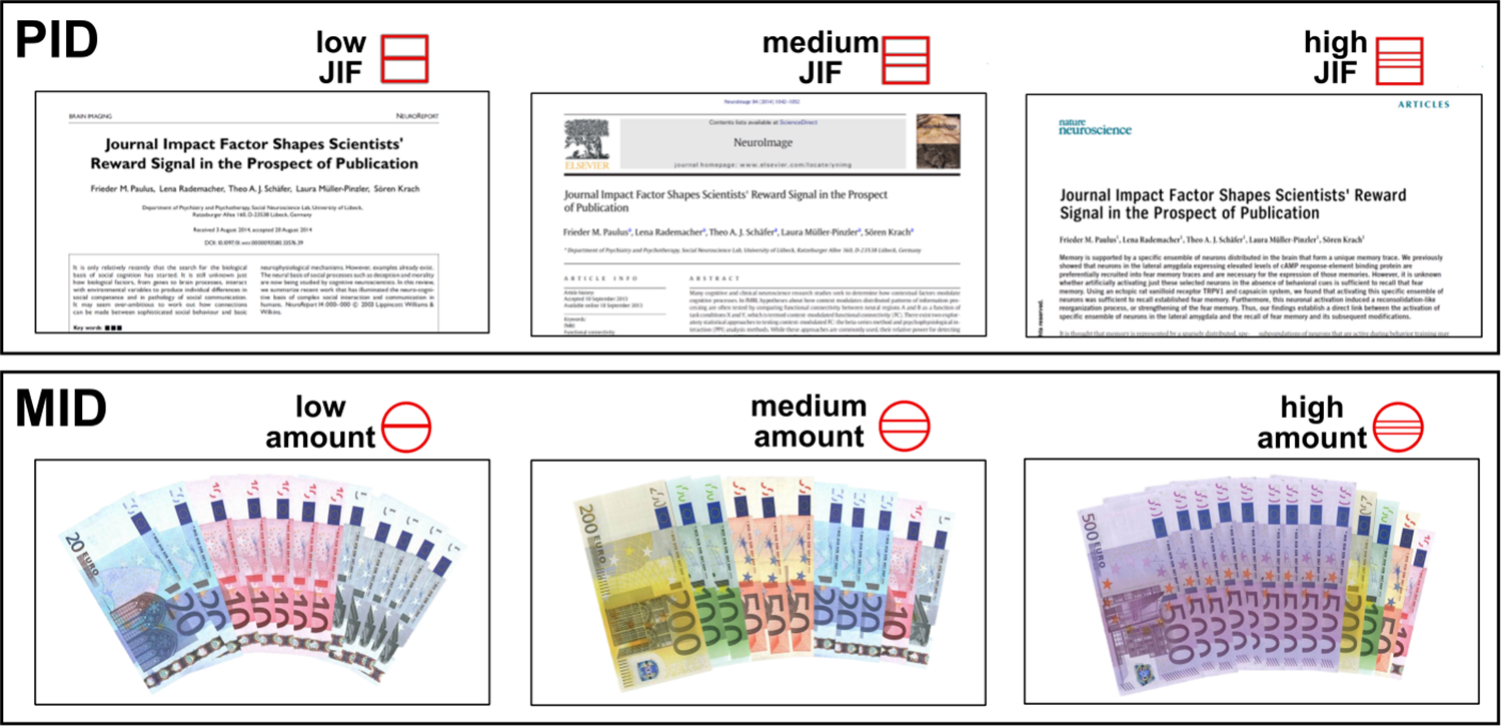
Example stimuli for each level for the publication (PID) and monetary incentive delay (MID) condition as used in study one. Images exemplify potential outcomes for each level of incentive together with the corresponding cues in the anticipation period in red symbols. Outcomes in the PID comprised personalized title pages of potential neuroscience journal publications with different journal impact factors (JIF, see methods). The title pages as used in the here presented figures were created for displaying purposes only.

In study one, we compared the MID to the PID. Thereby the JIFs of the potentially presented manuscripts were selected to vary between “low”, “medium”, and “high” and the monetary rewards varied accordingly with three levels between “low, “medium” and “high” amounts of money. In study two we only used potential publications in those journals of the “high” impact condition but varied the authorship position of the participant between being “first author” or “co-author”. In both studies, we also had a “no outcome” condition using scrambled pictures (see Fig 1).

In all trials, the potential outcome depended on the participants’ ability to press a button in time when a target symbol (filled red square) appeared on the screen. The task difficulty was thereby standardized for all participants by adjusting the target duration to each individual’s mean reaction time and adding 10% on top of this value. The individual reaction time was assessed based on a simple reaction time task which was conducted prior to the experiment (see [18], for a detailed description). In study one, the cues preceding the target symbol either signaled a potential reward outcome (denoted by circles for money incentives or squares for paper incentives) or “no outcome” in neutral trials (*n* = 20 trials; denoted by a triangle). The level of the potential reward was indicated by the number of horizontal lines in the cue. In the low JIF condition potential articles in journals such as “NeuroReport” were presented as outcomes (square with one horizontal line), in the medium JIF condition articles appeared in journals such as “NeuroImage” (square with two horizontal lines), and in the “high” impact condition articles were presented as if they were published in journals such as “Nature Neuroscience” (square with three horizontal lines). For each level of publication incentive eight journals were chosen and two to three different title pages were created for each journal and participant to get *n* = 20 unique stimuli for each level of reward. On average, the 2013 JIF of the journals in the “low” condition was *M* = 2.38±0.69, *M* = 6.22±1.21 in the “medium” condition, and *M* = 21.02±8.47 in the “high” condition. The monetary incentives in the three levels of the MID were generated to match the levels of JIFs in the PID conditions. To do so, a sample of ten senior neuroscientists (mean age: 36.3±6.60 years, 5 female) estimated the value of a publication in the selected journals in monetary units prior to this study. The corresponding monetary units were *M* = 214±74€ in the “low” condition, *M* = 766±162€ in the “medium” condition and *M* = 3,793±1,742€ in the “high” condition. For each journal a picture of a set of Euro bills was arranged so that the sum of the bills matched the money equivalent to the selected journal (see Table 1). To achieve *n* = 20 rewarding stimuli for each level, the monetary rewards were each presented two or three times (see Fig 1).

**Table 1:** Selected Journals, Journal Impact Factors (JIFs), and Money Equivalents. *Note*. *M* = mean; *SD* = standard deviation. The rated JIF represents the average of the participants’ guesses of the JIF of each journal and assessed after the post-experimental evaluation of the reward value of the stimuli.

Journal		2013 JIF	Rated JIF		Monetary Equivalent
			*M*	*SD*	
High JIF					
	Science	31.48	24.00	9.75	6,880
	Nature Reviews Neuroscience	31.38	17.49	9.32	5,260
	Nature Medicine	28.05	15.74	6.93	4,860
	Annual Reviews Neuroscience	22.66	11.52	8.08	2,720
	Neuron	15.98	11.64	5.12	2,560
	Nature Neuroscience	14.98	14.97	5.96	4,520
	Trends in Neuroscience	12.90	7.75	4.91	1,970
	Nature Communications	10.74	13.72	7.14	1,570
Medium JIF					
	Cerebral Cortex	8.31	6.87	2.52	940
	Human Brain Mapping	6.92	6.26	2.40	835
	Journal of Neuroscience	6.75	7.48	4.08	1,000
	Philosophical Transactions of The Royal Society B: Biological Sciences	6.31	8.14	5.71	700
	NeuroImage	6.13	6.36	1.58	890
	Cortex	6.04	6.61	3.50	625
	Journal of Cognitive Neuroscience	4.69	5.26	1.15	545
	Brain Structure Function	4.57	4.12	1.75	590
Low JIF					
	Neuropsychologia	3.45	3.81	1.74	375
	BMC Neuroscience	2.85	3.64	2.17	190
	Brain Research	2.83	3.83	1.77	210
	Brain and Cognition	2.68	4.06	1.07	220
	Experimental Brain Research	2.17	3.29	1.67	270
	Neuroscience Letters	2.06	3.77	4.28	160
	Neuroreport	1.64	3.77	3.46	115
	Neurocase	1.38	2.58	2.16	170

**Fig 2:**
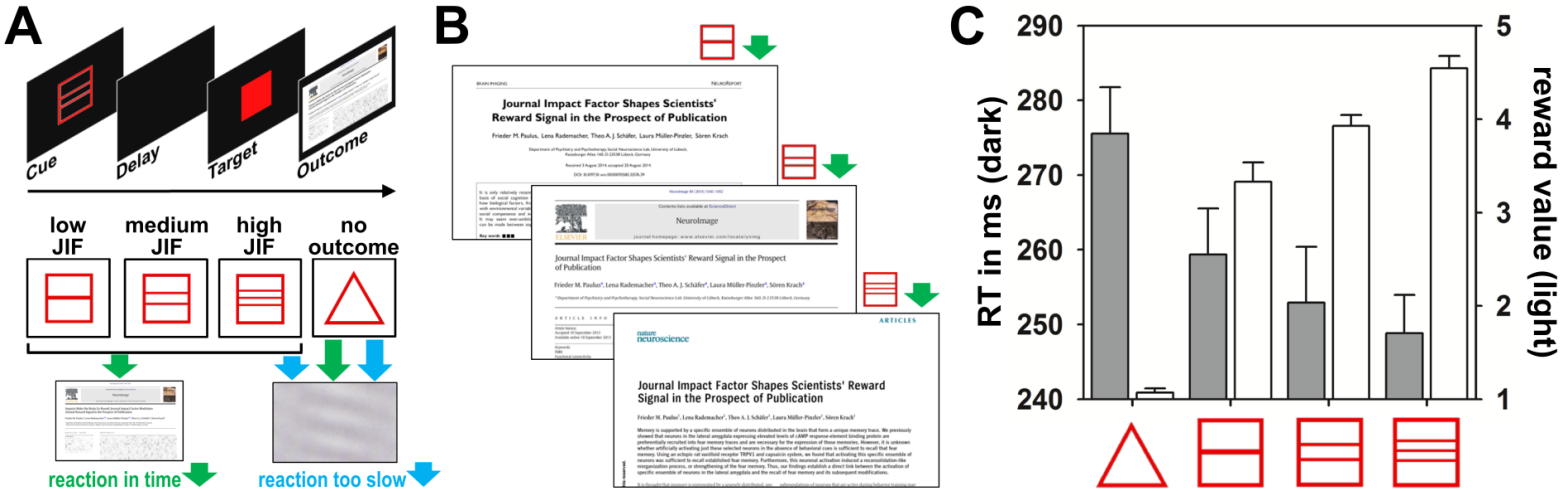
Design, stimuli and behavioral results of the publication incentive delay (PID) task in study one. **A**. Trial sequence of the functional magnetic resonance imaging (fMRI) paradigm together with cues and potential outcomes. The anticipation period lasted 1,000 ms, followed by a delay period for a variable length of time (between 2,250 and 2,750 ms) and the target (individually adjusted between 209 and 300 ms). Outcomes were presented for 4,000 ms starting 300 ms after target onset. **B.** Example stimuli for each level of the PID condition. **C**. Mean and standard errors of the reaction times and reward values for each level of publication incentive. Test for linear trends revealed significant decrease in reaction time (*F*(1,17) = 39.54, *p* < .001) and significant increase in reward value (*F*(1,17) = 49.07, *p* < .001) with higher levels of the journal impact factor (JIF).

Participants were asked to respond as fast as possible to all cues. Successful responses were acknowledged by presenting a picture of the corresponding title page of a potential journal publication or a picture of the respective amount of money in Euro bills. If participants failed to react in time or in neutral trials, a scrambled picture was presented (see Fig 2).

In study two, we implemented a similar version of the PID but varied the author's position while keeping the JIF of the publication constant. The cues preceding the target therefore either indicated to receive a first author position on a publication in a high-impact journal (*n* = 20; denoted by a ∩) or a co-author position on a publication in a high-impact journal (*n* = 20; denoted by a U) as outcome. In the neutral trials (*n* = 20; denoted by a triangle) no outcome could be gained. As in study one, the similar eight high impact journals were chosen and two to three distinctive title pages were created for each journal per author position and participant.

The trial structure was similar in both experimental paradigms. Each trial started with the presentation of one of the cues for 1,000 ms (anticipation period), followed by a delay period for a variable length of time (jittered between 2,250 and 2,750 ms) and the target (individually adjusted between 209 and 300 ms). The feedback with the outcome of the last trial was presented for 4,000 ms (consummatory period) starting 300 ms after target onset. Trial conditions were pseudo-randomly ordered with inter-trial-intervals jittered between 2,500 and 3,000 ms. With a total of 140 trials (i.e. MID = 60; PID = 60; neutral = 20) the first experimental paradigm added up to a total of 1,645 seconds and the 60 trials of the second experiment (first author = 20; co-author = 20; neutral = 20) to 699 seconds.

After providing written informed consent, all participants were carefully instructed about the experimental procedure and the purpose of the study. Since participants were neuroscientists, they were familiar with the principles of fMRI scanning and the nature of the incentive delay paradigm. Before practice, participants completed the reaction time task to adjust the task difficulty. Prior to entering the scanner they then performed a practice session comprising trials for both tasks to familiarize themselves with the experiment. In the fMRI, participants first completed the task comparing the PID with the MID (study one) and afterwards performed the authorship position PID (study two). After scanning, in a post experimental examination participants were asked to rate their experienced reward for the different incentives of both experimental paradigms. One stimulus randomly chosen from the different title pages for each of the journals (*n* = 24 for the first study, 8 journals in each condition; and *n* = 16 for the second study, 8 for each first and co-author condition) and the distinctive amounts of money (*n* = 24) were presented, together with the scrambled neutral stimuli. The intensity of the subjective reward value for each of the presented stimuli was rated on a Likert scale ranging from 1 (‘not at all’) to 5 (‘very strong’). After the assessment of the reward value participants were asked to estimate the actual JIF of the presented journals (see Table 2). Finally, participants completed a questionnaire assessing socio-demographic variables.

**Table 2:** Reaction Times, Ratios of Hits, and Subjective Reward Value for the Monetary Incentive Delay (MID) and the Publication Incentive Delay (PID) Conditions in Study 1 and Study 2. *Note*. *M* = mean; *SE* = standard error. Reaction times and ratios of hits were assessed during the functional magnetic resonance imaging paradigm and the reward value of the stimuli was examined with a post-experimental questionnaire.

		Reaction Time		Ratio of Hits		Reward Value	
		*M*	*SE*	*M*	*SE*	*M*	*SE*
**Study 1: Monetary (MID) and Publication Incentive Delay (PID)**							
	no outcome	275.54	6.20	0.34	0.05	1.07	0.05
MID							
	low	269.01	6.62	0.44	0.04	2.26	0.19
	medium	262.07	7.05	0.51	0.04	3.12	0.20
	high	261.05	5.75	0.54	0.05	4.05	0.20
PID							
	low	259.37	6.16	0.54	0.04	3.33	0.21
	medium	252.90	7.49	0.58	0.04	3.93	0.12
	high	248.83	5.12	0.66	0.05	4.55	0.13
**Study 2: First and Co-Author PID**							
	no outcome	285.04	8.14	0.32	0.05	1.00	0.00
	first author	256.20	6.40	0.56	0.05	4.61	0.13
	co-author	259.25	5.75	0.54	0.05	3.93	0.18

### Data Acquisition

Participants were scanned at 3T (Siemens Trio, Erlangen) with 36 near-axial slices and a distance factor of 10% providing whole brain coverage. An echo planar imaging (EPI) sequence was used for acquisition of functional volumes during the experiment (TR = 2.2 s, TE = 30 ms, flip angle = 90°, slice thickness = 3 mm, FoV = 192). Stimuli were presented on an LCD screen with Presentation 11.0 software package (Neurobehavioral Systems, Albany, CA, USA). A total of 760 volumes were acquired in study one and a total of 330 functional volumes were acquired in study two.

### Data Analysis

#### Behavioral Data

Data were analyzed with PASW Statistics 18 (SPSS, 2009, Chicago, IL). Each participant’s reaction times, ratios of hits and reward value as assessed after scanning were averaged within each level of incentives and the neutral condition for the first and second study. Reaction times were included for reactions from 50 ms to 500 ms after target onset, containing those for misses. Averaged variables were then analyzed using separate ANOVAs for PID, MID and the first and co-author PID paradigm with the incentive level as a within-subject factor. For MID (“low”, “medium”, “high”, and “no outcome”) and PID (“low”, “medium”, “high” and “no outcome”) each factor had four levels and for the second study the within-subject factor had three levels (“first author”, “co-author”, and “no outcome”). A priori defined contrasts for linear increase or decrease across the factor levels in the MID and the PID and comparing the first author vs the co-author were tested.

#### Functional MRI Data

FMRI data were analyzed using SPM8 (www.fil.ion.ucl.ac.uk/spm). The first seven images of each run were dummy scans and were discarded from further analyses. The remaining 753 EPI volumes for the first run and 323 EPI volumes for the second run were corrected for timing differences of the slice acquisitions, motion-corrected and spatially normalized to the standard template of the Montreal Neurological Institute (MNI) using the EPI template. The normalized volumes were resliced with a voxel size of 2x2x2 mm, smoothed with an 8 mm full-width half-maximum isotropic Gaussian kernel and high-pass filtered at 1/128 Hz to remove low frequency drifts.

Statistical analysis was performed in a two-level, mixed-effects procedure. For the first run the fixed-effects generalized linear model (GLM) on the first level included three epoch regressors modeling hemodynamic responses to the anticipation of incentives for the MID (3), three regressors modeling the anticipation of incentives for the PID (3), one for the anticipation of no outcome (1), and one for the consumption phase (1) with the abovementioned stimulus durations. Six additional regressors modeling head movement parameters were introduced to account for noise. The fixed-effects GLM for the second paradigm included two regressors modeling the anticipation of the two different author positions (2), one regressor for the anticipation of no outcome (1), one for the consumption phase (1), and six regressors for the head movement parameters.

For the random-effects GLM on the group-level all contrasts of interest were calculated on the first-level and the respective contrast images of activation differences were then analyzed on the group-level with one sample *t*-tests or paired sample *t*-tests. Linear increases in activation with increasing anticipated reward value across all four incentive levels for PID and MID were analyzed within a nucleus accumbens (NAcc) region-of-interest (ROI) which was derived from an automated meta-analysis of 143 studies (http://www.neurosynth.org). Based on textmining, meta-analysis, and machine learning techniques the Neurosynth framework generates probabilistic maps for cognitive constructs based on forward or reverse inference statistics [23]. For the means of the present analyses we computed a reverse inference map for the term ‘nucleus accumbens’ and thresholded the resulting Z-map at Z≥10. These procedures ensured a sufficiently smooth but specific ROI for the bilateral NAcc. To correlate the NAcc’s signal with the pJIF and for displaying purposes of the activation within each condition we extracted the average NAcc parameter estimates for the linear increase in the PID of study one for each participant. Results of the ROI analyses were thresholded at *p* < .05 applying family wise error (FWE) correction based on Gaussian random-field theory as implemented in SPM8.

To examine the raw BOLD signal changes in the NAcc after cue onset we used MarsBaR v 0.43 [24]. We defined a peristimulus interval of 15 seconds with a time resolution of one second. For displaying purposes, the interval for the expected peak was then estimated based on the canonical hemodynamic response function as specified in SPM8. For each bin we tested the effects of interest separately with one-sample *t*-tests.

## Results

As expected, neuroscientists showed greater goal-directed behavior in the expectation of high JIF publications. Reaction times decreased with greater JIF (*F*(1,17) = 39.54, *p* < .001) and hit ratios increased accordingly (*F*(1,17) = 49.07, *p* < .001). Even within the context of this experimental paradigm, the JIF modulated goal-directed behaviors and was also related to the subjective value of the rewarding stimuli as assessed after the experiment (*F*(1,17) = 486.98, *p* < .001, for additional behavioral data see Table 2 and S1 File).

**Fig 3:**
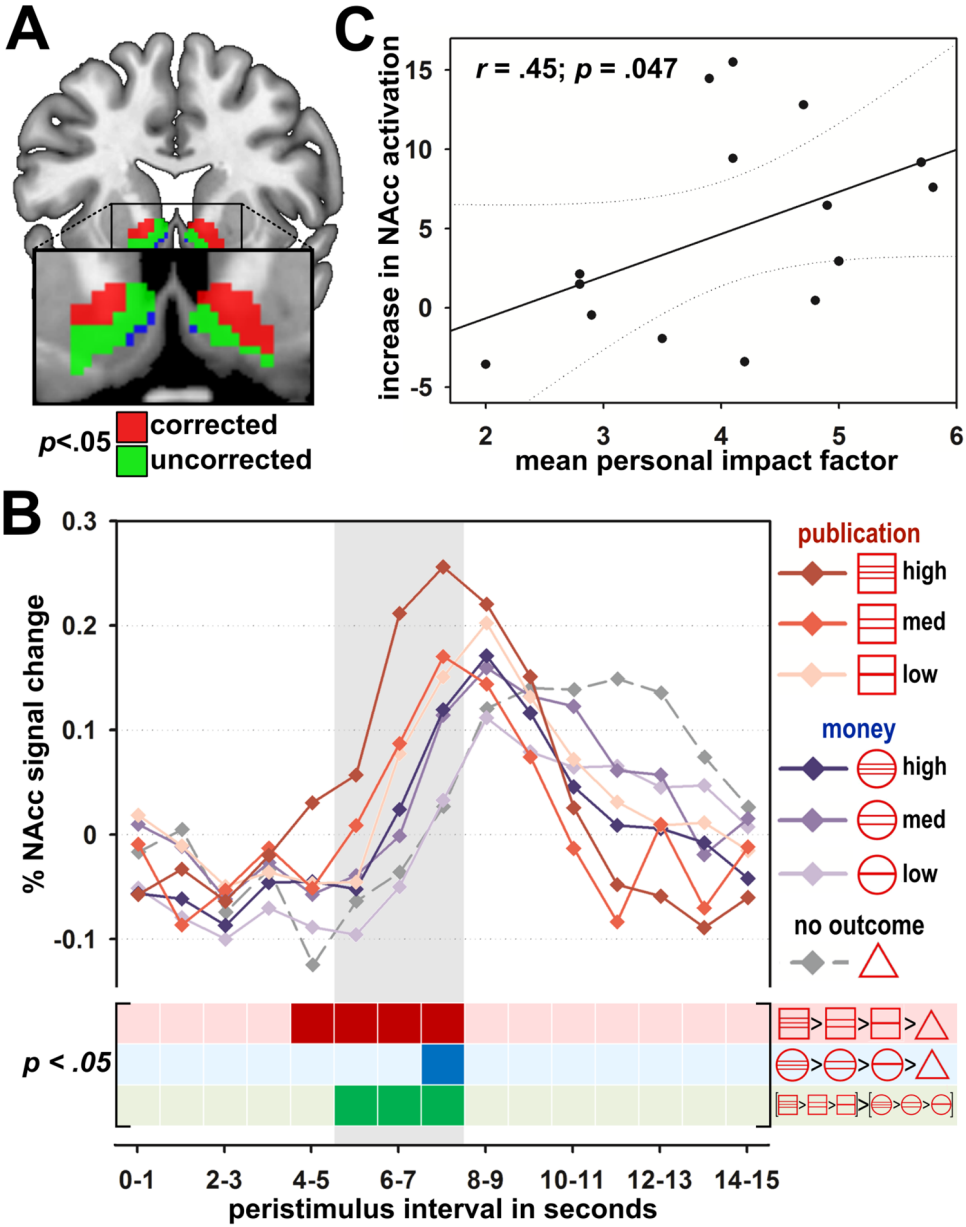
Neuroimaging findings of the publication incentive delay (PID) condition in study one and comparison to the monetary incentive delay (MID) condition. **A.** Voxels within the nucleus accumbens (NAcc) that show significant effects for the linear increase in activation with increasing journal impact factor during anticipation of publications. Family-wise error corrected voxels are displayed in red (left NAcc: *t*(17) = 3.75, *p* = .024; right NAcc: *t*(17) = 4.15, *p* = .009). Voxels that are significant at uncorrected thresholds are displayed in green. Blue voxels show those voxels within the mask that had no significant effect. **B.** Mean percent NAcc BOLD signal change after cue onset. The grey shaded area indicates the expected interval for the peak of the cue related BOLD response. BOLD signal changes in response to cues indicating title pages of journal publications are depicted in red colors and BOLD signal changes in response to the cues indicating monetary incentives in blue colors. The cue indicating no outcome is represented by the dashed grey line. The bottom area indicates the statistical significance of the contrast for the linear increase in BOLD signal change with higher level of incentive and the respective comparison between the PID and the MID condition for each bin. Significant bins at *p* < .05 are depicted with dark colors. **C.** Correlation of journal impact factor (JIF) associated increase in NAcc activation during the anticipation phase with mean personal JIF (pJIF) of the participating neuroscientists’ prior publication records.

These behavioral effects mapped very well to the reward signal of the bilateral NAcc: With the anticipation of higher JIF publications, the bilateral NAcc response increased and was more positive compared to lower JIF publications (left NAcc: *t*(17) = 3.75, *p* = .024; right NAcc: *t*(17) = 4.15, *p* = .009; corrected, see Fig 3; for results of whole-brain analyses of the PID, see Table 3, and of the MID, see Table A in S1 File), and the behavioral effects were correlated with the increase in the bilateral NAcc activity (reaction time: *r* = -.52, *p* = .013, reward value: *r* = .43, *p* = .038). Thus, the JIF as a novel and powerful paradigm in academia has already shaped the neural architecture of reward processing in science. This is corroborated by the finding of a stronger NAcc modulation with JIF as compared to monetary reward, which we examined as a baseline for control (right NAcc: *t*(17) = 3.51, *p* = .030, corrected). This suggests that publications and the respective JIF, as the new currency in science, are complementarily processed as monetary incentives with their long history as a reward in society. However, we cannot rule out that scientists do translate the JIF and high-impact publications into monetary units, as they indirectly contribute to financial success in the long run (salary and PI positions see [2]).

**Table 3:** Brain Areas with Stronger Activation During the Anticipation of Higher Journal Impact Factor (JIF) Publications. *Note*. Results refer to *p* < .001 for a whole-brain analysis and survive correction for multiple comparisons at cluster-level. MNI coordinates represent the peak voxel for each (sub-)cluster. The Cyto area column indicates the assigned cytoarchitectonical area as indicated by the SPM ANATOMY toolbox v1.8 if available (Eickhoff et al. 2005). Anatomical labels were derived respectively if available.

Anatomical Region		Cyto Area	Side		Cluster Size	MNI Coordinates			*t*	*p*	
						x	y	z			
**Linear Increase in Publication Rewards**											
Middle Occipital Gyrus			R		21,809	34	-90	20	10.79	<.001	
	Inferior Occipital	hOC4v (V4)	L			-28	-78	-10	10.44		
	Superior Occipital	Area 18	L			-16	-100	16	10.34		
Midbrain			R		4,173	2	-28	-6	7.38	<.001	
	Thalamus	Th-Temporal	R			2	-14	0	6.71		
	Midbrain		R			10	-14	-10	6.09		
Insula Lobe			L		305	-32	26	8	5.36	.043	
Precentral Gyrus		Area 4a	R		810	38	-12	42	5.35	<.001	
	Middle Frontal Gyrus		R			52	0	52	5.06		
	Precentral Gyrus		R			36	-2	50	5.00		
Rolandic Operculum			L		442	-38	-34	22	4.78	.011	
	Supra Marginal Gyrus	OP1	L			-48	-24	24	4.55		
	Postcentral Gyrus	OP1	L			-54	-18	18	4.40		
**Linear Increase in Publication Rewards > Linear Increase in Money Rewards**											
Midbrain			R	398	4	-28	-8	6.06	.012	
	Thalamus	Th-Prefrontal	R		6	-18	-2	5.40	
	Thalamus	Th-Prefrontal	R		12	-12	2	4.14	
Cuneus			L	361	-16	-78	16	5.68	.017
	Calcarine Gyrus		L		-22	-66	12	4.28	
SMA		Area 6	L	378	-6	2	52	5.25	.015
	Middle Cingulate Cortex		R		10	8	42	4.59	
	SMA	Area 6	L/R		0	-2	68	3.71	
Calcarine Gyrus			R	343	20	-70	14	4.96	.021
	Calcarine Gyrus		R		24	-56	10	4.86	
	Calcarine Gyrus		R		18	-80	16	4.47	

The findings within the current experiment were related to previous publishing performance of the involved neuroscientists. The modulation of the NAcc response by the JIF was positively correlated with the scientist`s mean personal JIF (pJIF, *r* = .45, *p* = .047, see Fig 3). The pJIF was computed based on the publications of each neuroscientist (www.pipredictor.com, [2]). This means that scientists who, on average, published their work in higher-ranking journals had a stronger increase in NAcc activation in the anticipation of higher JIF publications. Incorporation of the JIF into the reward system thus seems to be related to goal-directed behavior during the publication process. However, we are unable to decide whether publication in higher-impact journals is the cause or consequence of the reward signal in the NAcc. On the one hand, it is possible that past experiences with publication in higher-impact journals shaped the sensitivity of the reward circuits to this measure. On the other hand, it might be the case that greater reward signaling of the NAcc, as a motivational force, led to more effort and persistence in order to achieve a higher JIF for one’s findings.

**Fig 4:**
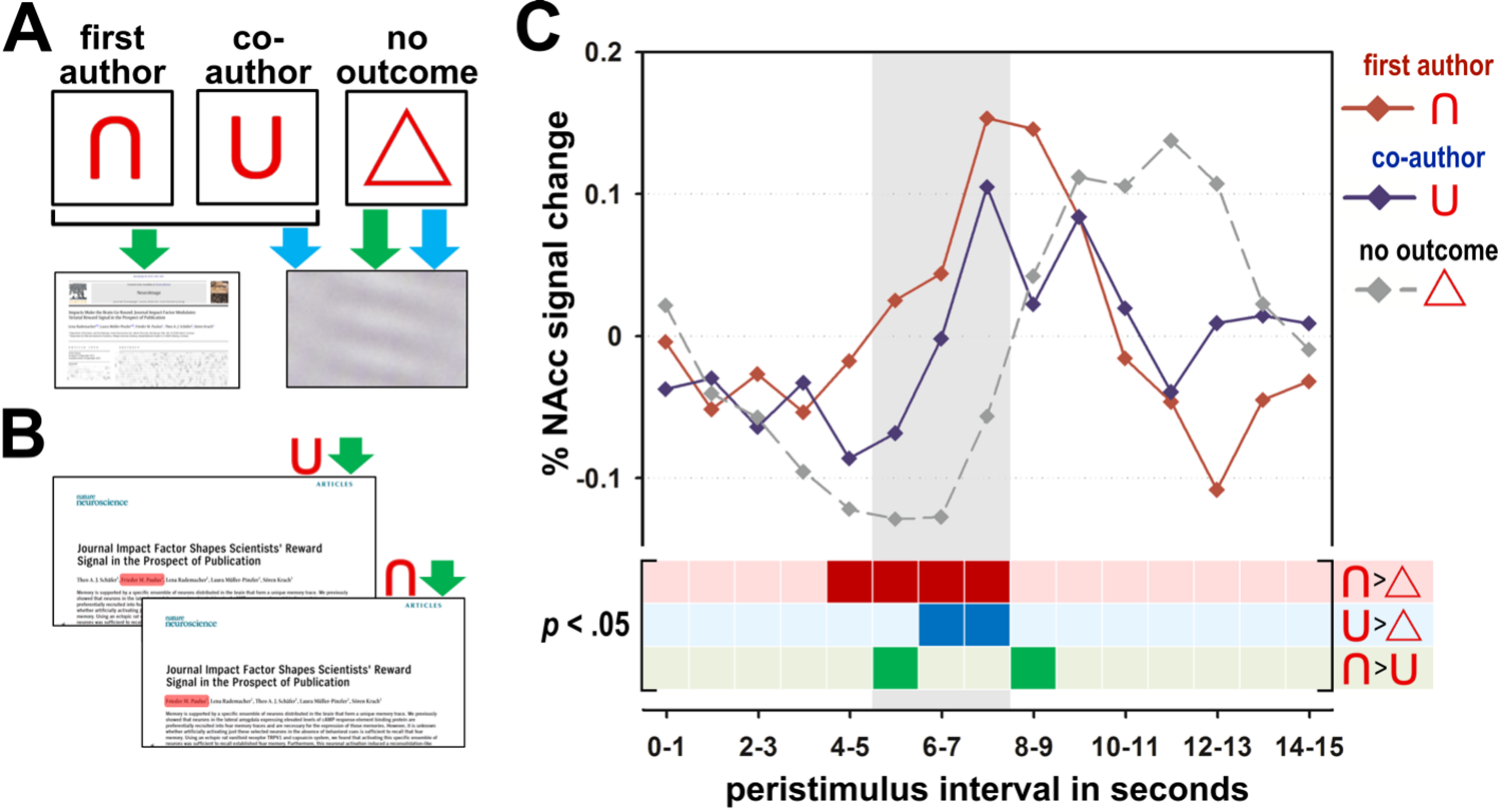
Cues, stimuli and results of the first and co-author incentive delay task in study two. **A.** Cues and potential outcomes. **B.** Example stimuli for either a first or co-authorship in a high-impact journal. For displaying purposes the author’s position is highlighted in red. **C.** Percent NAcc BOLD signal change after cue onset. The grey shaded area indicates the expected interval for the peak of the cue related BOLD response. BOLD signal changes in response to the cue indicating title pages of a first author publication as potential outcome are depicted in red and BOLD signal changes in response to the cue indicating a co-author position in blue. The cue indicating no outcome is represented by the dashed grey line. The bottom area indicates the statistical significance of the respective comparison of BOLD signal changes to the different cues for each bin. Significant bins at *p* < .05 are depicted with dark colors.

The authorship position had a similar impact on the anticipatory reward signal. The results of study two show that the same brain regions that were sensitive to the JIF also had a stronger signal during anticipation of first as compared to co-authorships (right NAcc: *t*(17) = 4.94, *p* = .003, corrected, see Fig 4 and see Table B in S1 File for the average effect of anticipation of first- and co-authorship publications).

**Fig 5:**
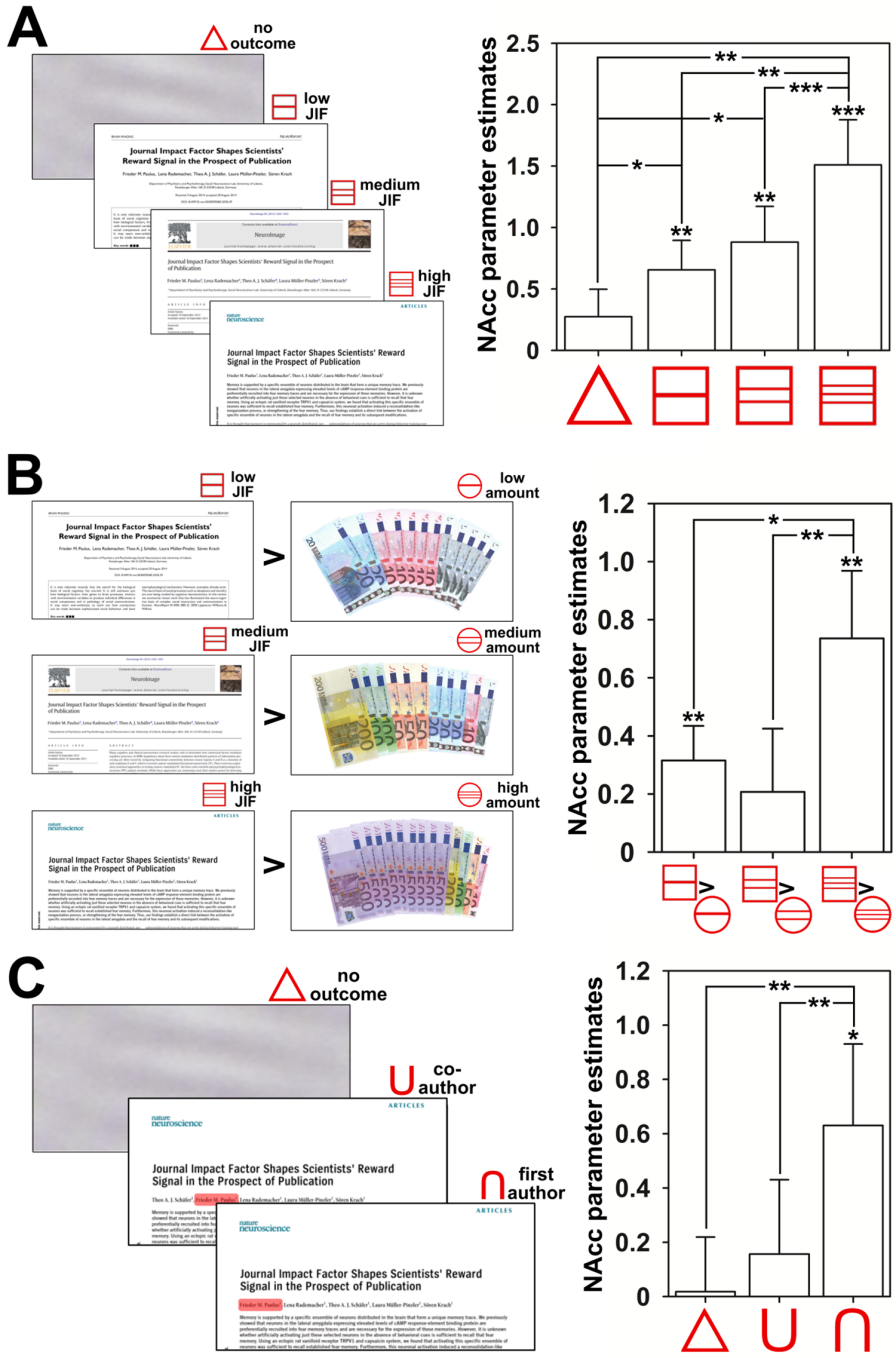
Summary of the NAcc parameter estimates for the anticipation of publication and monetary incentives. **A**. NAcc responses during the publication incentive delay condition (PID) for the three different levels of anticipated journal impact factors (JIF). Cues during anticipation (red symbols) are presented with an example for the potential outcome (left side). The bar chart depicts the means and standard errors (SEM) of the estimated NAcc hemodynamic signal in response to the different cues (right side). Parameter estimates for the hemodynamic response during anticipation were computed as averages within the bilateral NAcc mask. **B.** Stronger NAcc responses during the publication incentive delay (PID) compared to the monetary incentive delay (MID) conditions. The contrasts for each level of comparison are depicted with the cues and the corresponding outcomes in the PID and MID (left side). The bar chart illustrates the means with the SEMs of the differences in the estimated NAcc hemodynamic signal during the anticipation of corresponding levels of the MID and PID (right side). **C.** NAcc responses during the anticipation of publications with either a first or a co-author contribution in high-impact journals. Cues during anticipation are presented with an example for the corresponding outcome in the first- and co-author condition (left side). The bar chart depicts the means and SEMs of the estimated NAcc hemodynamic signal in response to cues indicating a first- or co-authorship (right side). Please note that asterisks above the SEM indicators represent significant NAcc effects for the corresponding contrast as estimated with a one-sample *t*-test (one-sided) and asterisks between bars represent significant differences between contrasts as estimated with a paired-sample *t*-test (one-sided) with * *p* < .05, ** *p* < .01 and *** *p* < .001.

In this line, the subjective assessment of the reward value as obtained after the experiment was also higher for first compared to co-authorships (*F*(1,17) = 16.03, *p* = .001; see S1 File for more detailed information). A scientist’s NAcc is thus sensitive to both aspects that are usually considered for estimating the value of a publication, the JIF and the authorship position, and motivates goal-directed behavior if a relevant stimulus is within reach (for a summary of the NAcc’s parameter estimates of both experiments, see Fig 5).

## Discussion

According to some economic theories, the human being, viewed as homo economicus, is guided by the expectation that the benefits of one’s actions outweigh the costs. For scientists, the costs of their behavior are significant, with often uncertain outcomes. The results of this study show how scientists have adapted to the predominant reward structure in the environment and have incorporated the currently prevailing paradigm of the scientific community to “publish [in high-impact journals] or perish” in order to guide behavior.

From a neuroscience perspective, this study offers novel insights by providing first empirical evidence that the JIF and the authorship position do actually influence human behavior and neural response patterns. Even though the impact of the JIF has been extensively discussed, with first empirical data showing that it influences institutional decision making in academia, nothing was known about how it influences scientists’ motivation. Through this experiment, albeit in a very controlled laboratory setting, we now have an idea of how deeply entrenched the concept of the JIF has become on the neural systems level. We find consistent indications that a higher JIF is associated with greater subject reward value, faster reaction times, and stronger NAcc response in the prospect of publication.

What do these findings tell us about mesolimbic dopamine signaling and the role of the NAcc in general? The NAcc is involved not only in reward anticipation but also in locomotion [25], and anticipatory responses have been shown to reflect both the reward value and the subsequent motor effort [26]. Thus, it might be difficult to dissociate motivation and action, also in the current experiment. However, we tentatively propose that the correlation described here of NAcc activation with a complex outcome measure of motivated behavior, the pJIF, provides an external validation of the striatal activation, as a motivational signal, using real-life data. This is particularly important as previous measures for validation have been restricted to reaction times and hit rates in the same experiment, which are also corroborated by the present study. Regardless of the potential ambiguity of the NAcc response, this study provides a very illustrative example of plasticity in striatal reward processing by showing that even very arbitrary measures can be constructed to guide behavior–if properly curated as incentives.

One might question whether the presented studies are suitable to draw such far reaching conclusion. First, the experimental paradigms were designed in a way that participants received virtual incentives instead of actual monetary gains and real publications in the depicted journals. However, at present this is impossible to realize for obvious reasons. Second, the sample might not be representative for the whole scientific community and effects might vary between research disciplines and the publication culture of specific departments. Future studies could pick up on this point and examine how scientists differently cope with the urge for publication and the pressure to publish in their disciplines. In addition, one should keep in mind that the current sample comprised relatively young neuroscientists, who are in the early stages of their career. At this point in the career, first-author positions have the highest relevance for academic success [2]. It is therefore probable that the significance of the authorship position changes in the course of one’s scientific career, and (successful) senior scholars who have built their own group might increasingly value a last-authorship over a first-authorship.

Nevertheless, from a broader perspective, our study demonstrates how the reward system sensitively adapts to the new and upcoming paradigms in society, and helps to provide an understanding of persistence and success in a highly competitive environment. The implications of this specific behavioral adaption within the ecological niche of the scientist’s habitat remain unknown, and may also have effects which were not intended by the scientific community [9]. The excessive use of the JIF as the predominant incentive and indicator for scientific quality might be dysfunctional in motivating scientists to “publishing well rather than often” [27]. Further, the extensive use of extrinsic rewards such as money, but also surrogates such as the JIF, could compromise intrinsic motivation and curiosity [28]. It is intriguing to recognize that most of the criticism of the JIF is about finding “better measures in town” to quantify scientific excellence, whereas relatively little attention is devoted to thinking about the general implications of using these metrics. Accordingly, we wish to close with a reference to Werner’s recent comment that “[m]any negative effects of bibliometrics come not from using it, but from the anticipation that it will be used. When we believe that we will be judged by silly criteria, we will adapt and behave in silly ways” [5].

## Supporting Information

S1 File Supporting Behavioral and Neuroimaging Results. Increased Activation with Linear Increase in Anticipated Monetary Rewards (Table A). Increased Activation during Anticipation of First and Co-Authorships compared to No Outcome (Table B).
(DOC)

S1 Note Criteria of “scientific excellence”. (DOC)
