# Supplementary material for: Journal Impact Factor Shapes Scientists’ Reward Signal in the Prospect of Publication
Source: PLoS One. 2015 Nov 10;10(11):e0142537. doi: 10.1371/journal.pone.0142537 (PMC4640843; doi:10.1371/journal.pone.0142537)
Supplement: S1 File — Increased Activation with Linear Increase in Anticipated Monetary Rewards (Table A). Increased Activation during Anticipation of First and Co-Authorships compared to No Outcome (Table B). (DOC) [file pone.0142537.s001.doc]

Journal Impact Factor Shapes Scientists’ Reward Signal in the Prospect of Publication

Frieder Michel Paulus, Lena Rademacher, Theo Alexander Jose Schäfer, Laura Müller-Pinzler, Sören Krach

**S1 File. Supporting Behavioral and Neuroimaging Results.**

**Behavioral Data**

ANOVAs revealed a significant main effect for the level of incentive in the reaction time (*F*(3,51)=6.21, *p*=.001), ratio of hits (*F*(3,51)=11.74, *p*<.001), and reward value (*F*(3,51)=121.63, *p*<.001) in the MID of study one. For the PID, main effects for the level of incentive were also significant for reaction time (*F*(3,51)=17.45, *p*<.001), ratio of hits (*F*(3,51)=19.40, *p*<.001), and reward value (*F*(3,51)=154.64, *p*<.001). The first and co-author PID in study two revealed similar main effects in the reaction time (*F*(3,51)=10.93, *p*<.001), ratio of hits (*F*(3,51)=25.43, *p*<.001), and reward value (*F*(3,51)=374.07, *p*<.001).

Post-hoc contrasts for the linear change with increasing anticipated reward outcomes indicated a significant decrease in reaction times for MID (*F*(1,17)=17.11, *p*=.001) and PID (*F*(1,17)=39.54, *p*<.001). The ratio of hits showed a similar effect for the linear change, however, they increased with greater anticipated reward outcomes for MID (*F*(1,17)=17.74, *p*=.001) and PID (*F*(1,17)=49.07, *p*<.001) and the reward value of the stimuli, as assessed after scanning showed a significant effect in the same direction (MID: *F*(1,17)=208.29, *p*=.001; PID: *F*(1,17)=486.98, *p*<.001). There was no significant difference in the reaction time (*F*(1,17)=0.95, *p*=.343) and ratio of hits (*F*(1,17)=0.40, *p*=.537) between the first author and co-author condition, but the reward experience in response to the incentives was significantly stronger in the first compared to the co-author condition (*F*(1,17)=16.03, *p*=.001).

**Table A*.* Increased Activation with Linear Increase in Anticipated Monetary Rewards.**

| Anatomical Region | | Cyto Area | Side | Cluster Size | MNI Coordinates | | | *t* | *p* |
| --- | --- | --- | --- | --- | --- | --- | --- | --- | --- |
|  |  |  |  |  | x | y | z |  |  |
|  |  |  |  |  |  |  |  |  |  |
| Superior Occipital Gyrus | | Area 18 | L | 9,905 | -16 | -102 | 12 | 10.77 | <.001 |
|  | Cuneus | Area 18 | R |  | 20 | -96 | 14 | 10.18 |  |
|  | Calcarine Gyrus | Area 17 | R |  | 18 | -98 | 4 | 9.83 |  |
| Thalamus | | Th-Visual | R | 879 | 24 | -28 | -4 | 7.72 | <.001 |
|  | Midbrain |  | R |  | 8 | -10 | -14 | 6.11 |  |
|  | Amygdala |  | R |  | 18 | 2 | -12 | 5.15 |  |
| Thalamus | | Th-Parietal | L | 276 | -20 | -24 | -6 | 6.53 | .018 |
|  | Thalamus | Th-Parietal | L |  | -24 | -24 | 8 | 5.62 |  |
|  | Thalamus | Th-Prefrontal | L |  | -18 | -20 | 12 | 5.07 |  |
| Postcentral Gyrus | | Area 6 | L | 1,201 | -44 | -24 | 64 | 6.50 | <.001 |
|  | Precentral Gyrus | Area 6 | L |  | -40 | -12 | 66 | 5.54 |  |
|  | Postcentral Gyrus | Area 4p | L |  | -30 | -26 | 52 | 5.21 |  |
| Inferior Frontal Gyrus | | | R | 359 | 42 | 12 | 8 | 6.09 | .005 |
|  | Putamen |  | R |  | 24 | 6 | 12 | 5.40 |  |
|  | Putamen |  | R |  | 32 | -6 | 10 | 5.20 |  |
| Putamen | |  | L | 502 | -28 | 6 | 6 | 5.64 | <.001 |
|  | Amygdala | Amyg (LB) | L |  | -20 | -6 | -18 | 5.12 |  |
|  | Pallidum |  | L |  | -20 | -2 | -4 | 4.76 |  |
| SMA | | Area 6 | L | 619 | -8 | -10 | 52 | 5.52 | <.001 |
|  | SMA | Area 6 | R |  | 8 | -6 | 60 | 5.12 |  |
|  | SMA | Area 6 | R |  | 2 | -10 | 66 | 4.91 |  |
|  |  |  |  |  |  |  |  |  |  |

*Note*. Results refer to *p*< .001 for a whole-brain analysis and survive correction for multiple comparisons at cluster-level. MNI coordinates represent the peak voxel for each (sub-)cluster. The Cyto area column indicates the assigned cytoarchitectonical area as indicated by the SPM ANATOMY toolbox v1.8 if available (Eickhoff et al. 2005). Anatomical labels were derived respectively if available.

**Table B. Increased Activation during Anticipation of First and Co-Authorships compared to No Outcome.**

| Anatomical Region | | Cyto Area | Side | Cluster Size | MNI Coordinates | | | *t* | *p* |
| --- | --- | --- | --- | --- | --- | --- | --- | --- | --- |
|  |  |  |  |  | x | y | z |  |  |
|  |  |  |  |  |  |  |  |  |  |
| Middle Occipital Gyrus | |  | L | 2,471 | -24 | -94 | 0 | 8.81 | <.001 |
|  | Inferior Occipital Gyrus | hOC4v (V4) | L |  | -36 | -8 | -86 | 8.37 |  |
|  | Fusiform Gyrus |  | L |  | -38 | -64 | -12 | 7.55 |  |
| Insula Lobe | |  | L | 685 | -30 | 28 | 2 | 7.78 | <.001 |
|  | Pallidum |  | L |  | -14 | 0 | -2 | 4.55 |  |
|  | Putamen |  | L |  | -20 | 4 | 8 | 4.27 |  |
| Inferior Occipital Gyrus | |  | R | 2,095 | 38 | -84 | -6 | 7.41 | <.001 |
|  | Middle Occipital Gyrus |  | R |  | 26 | -94 | 8 | 7.39 |  |
|  | Lingual Gyrus | Area 17 | R |  | 16 | -90 | -4 | 6.71 |  |
| Superior Parietal Lobule | | hIP3 | L | 1,070 | -28 | -54 | 50 | 6.83 | <.001 |
|  | Superior Parietal Lobule | SPL (7A) | L |  | -18 | -66 | 54 | 5.71 |  |
|  | Middle Occipital Gyrus |  | L |  | -26 | -74 | 26 | 5.03 |  |
| Precentral Gyrus | |  | R | 832 | 38 | 0 | 50 | 6.18 | <.001 |
|  | Middle Frontal Gyrus |  | R |  | 44 | 2 | 60 | 5.34 |  |
|  | Precentral Gyrus | Area 6 | R |  | 42 | -8 | 48 | 4.27 |  |
| Superior Frontal Gyrus | | Area 6 | L | 980 | -20 | -6 | 76 | 6.04 | <.001 |
|  | Precentral Gyrus | Area 6 | L |  | -46 | 0 | 54 | 5.36 |  |
|  | Precentral Gyrus | Area 6 | L |  | -52 | -4 | 48 | 5.29 |  |
| Insula Lobe | |  | R | 1,499 | 34 | 26 | 0 | 5.95 | <.001 |
|  | Thalamus | Th-Prefrontal | R |  | 16 | -6 | 0 | 5.22 |  |
|  | Midbrain |  | R |  | 4 | -30 | -6 | 5.00 |  |
| Angular Gyrus | | hIP3 | R | 1,223 | 30 | -60 | 50 | 5.81 | <.001 |
|  | Precuneus | SPL (7P) | R |  | 12 | -70 | 50 | 5.69 |  |
|  | Middle Occipital Gyrus |  | R |  | 32 | -66 | 26 | 4.39 |  |
| SMA | | Area 6 | L | 1,663 | -2 | 2 | 62 | 5.71 | <.001 |
|  | Middle Cingulate |  | R |  | 6 | 20 | 38 | 5.65 |  |
|  | SMA | Area 6 | L |  | -4 | 14 | 44 | 5.14 |  |
|  |  |  |  |  |  |  |  |  |  |

*Note*. Results refer to *p*< .001 for a whole-brain analysis and survive correction for multiple comparisons at cluster-level. MNI coordinates represent the peak voxel for each (sub-)cluster. The Cyto area column indicates the assigned cytoarchitectonical area as indicated by the SPM ANATOMY toolbox v1.8 if available (Eickhoff et al. 2005). Anatomical labels were derived respectively if available.
