# Supplementary material for: Journal Impact Factor Shapes Scientists’ Reward Signal in the Prospect of Publication
Source: PLoS One. 2015 Nov 10;10(11):e0142537. doi: 10.1371/journal.pone.0142537 (PMC4640843; doi:10.1371/journal.pone.0142537)
Supplement: S1 Note — (DOC) [file pone.0142537.s002.doc]

Journal Impact Factor Shapes Scientists’ Reward Signal in the Prospect of Publication

Frieder Michel Paulus, Lena Rademacher, Theo Alexander Jose Schäfer, Laura Müller-Pinzler, Sören Krach

**S1 Note. Criteria of “scientific excellence”**

Notably, there are tremendous efforts to find the “best measure” to describe scientific excellence. These consider evaluations on two levels: the level of a publication, which characterizes the impact of the presented findings in a broader sense, and the personal level, which assesses the accumulated impact of a scientist’s work in the field. Metrics regarding the evaluation of a single publication comprise, among other things, the number of citations, the number of reads or views, media outreach including tweets, and also more complex measures such as Altmetric, which integrates the different sources. A scientist’s “excellence” is quantified using composite measures such as the H-index, RG score, or Loop, which have different input variables, but mostly integrate the number of citations or the accumulated JIF.

An intriguing example regarding the entanglement of the JIF and the neurosciences can be found in the study by Behrens and colleagues [29]. Here, the authors did not use the JIF to determine the scientific excellence of a person or publication, but turned the tables, quantifying the scientific value of the subject of interest, i.e. regions within the brain. With their meta-analysis, they delineated regions that were more “fashionable” than others, because findings in these particular regions were published in journals with higher JIF. While this study provides an example from the field of neurosciences, other disciplines might be confronted with similar biases and certain topics which “sell” better than others.
